# Effect of interferon combined with ganciclovir on immune status in children with infectious mononucleosis

**DOI:** 10.12669/pjms.41.8.11331

**Published:** 2025-08

**Authors:** Jian Zhu, Haobin Song, Jingya Wang, Jing Bi, Yuanda Zhang

**Affiliations:** 1 Jian Zhu, Department of Clinical Laboratory, Hebei Key Laboratory of Infectious Diseases Pathogenesis, Precise Diagnosis and Treatment, Baoding, 071000 Hebei, China Baoding Hospital, Beijing Children’s Hospital Affiliated to Capital Medical University, Baoding, 071000 Hebei, China; 2 Haobin Song Department of Clinical Laboratory, Hebei Key Laboratory of Infectious Diseases Pathogenesis, Precise Diagnosis and Treatment, Baoding, 071000 Hebei, China Baoding Hospital, Beijing Children’s Hospital Affiliated to Capital Medical University, Baoding, 071000 Hebei, China; 3 Jingya Wang Department of Clinical Laboratory, Hebei Key Laboratory of Infectious Diseases Pathogenesis, Precise Diagnosis and Treatment, Baoding, 071000 Hebei, China Baoding Hospital, Beijing Children’s Hospital Affiliated to Capital Medical University, Baoding, 071000 Hebei, China; 4 Jing Bi, Department of Infectious Diseases, Hebei Key Laboratory of Infectious Diseases Pathogenesis, Precise Diagnosis and Treatment, Baoding, 071000 Hebei, China Baoding Hospital, Beijing Children’s Hospital Affiliated to Capital Medical University, Baoding, 071000 Hebei, China; 5 Yuanda Zhang, Department of Neurology, Hebei Key Laboratory of Infectious Diseases Pathogenesis, Precise Diagnosis and Treatment, Baoding, 071000 Hebei, China Baoding Hospital, Beijing Children’s Hospital Affiliated to Capital Medical University, Baoding, 071000 Hebei, China

**Keywords:** Human Interferon Alpha-1b, Infectious Mononucleosis, Immune Factors, Ganciclovir

## Abstract

**Objective:**

To investigate the effect of interferon combined with ganciclovir on immune status in children with infectious mononucleosis(IM) and explore the clinical efficacy.

**Methodology:**

This was a retrospectively study. A total of 90 children with IM admitted to Baoding Hospital, Beijing Children’s Hospital Affiliated to Capital Medical University from January 2022 to December 2023 were divided into the observation and control groups using a random number table. Afterward, the pre- and post-treatment humoral immunity indicators (IgG, IgA) and cellular immunity indicators (CD3+, CD4+, CD4+/CD8+) were compared between the observation and control groups.

**Results:**

IgG, IgA, CD3+, CD4+, and CD4+/CD8+ values in the observation group were significantly changed compared with those in the control group pre- and post-treatment, with statistical significance (p< 0.05); the post-treatment IgG, IgA, CD4+, and CD4+/CD8+ values in the two groups were significantly higher than the pre-treatment values, and CD3+ values were markedly lower than pre-treatment values, with statistical significance(p< 0.05); the post-treatment IgA and CD3+ values in the observation group were higher and lower than those in the control group, respectively, with statistical significance(p< 0.05).

**Conclusion:**

Human interferon α-1b combined with ganciclovir in the treatment of IM leads to faster subsidence of clinical manifestation. Meanwhile, IgA and CD3+ are sensitive experimental indicators with certain clinical significance in evaluating the effect of IM, which can be used as a reference for evaluating the efficacy of drugs and disease outcomes.

## INTRODUCTION

Infectious mononucleosis(IM) is caused by Epstein-Barr virus (EBV), with clinical manifestations of fever, sore throat, lymphadenopathy, tonsillar enlargement, etc. Children are at high risk of IM, and the virus continues to spread if not treated promptly, leading to various complications. Critically ill children even experience neurological manifestation, seriously affecting the normal development of children.^1^-^2^ Currently, IM is mainly treated with symptomatic and antiviral therapies, and there are no specific therapies available.

Despite being the predominant antiviral drug, clinical studies have found that it is difficult to achieve desired effects with ganciclovir alone.^3^ In this regard, human interferon α-1b has been shown to demonstrate better antiviral, anti-inflammatory, and immune function regulation effects and have a synergistic effect with ganciclovir, which enhances the inhibition of viral replication while promoting the recovery of immune system function and improving the body’s ability to resist viruses.^4^,^5^ In this study, human interferon α-1b combined with ganciclovir was utilized in the clinical treatment of 90 children with IM, so as to investigate its effect on the immune level of children and compare the therapeutic effects. The findings are reported as follows.

## METHODOLOGY

This was a retrospectively study. Ninety children with IM admitted to Baoding Hospital, Beijing Children’s Hospital Affiliated to Capital Medical University from January 2022 to December 2023 were divided into the observation and control groups using a random number table. According to the data of each indicator in the pre-survey, the sample size is estimated by 95% confidence interval, and the largest one is the sample size of the study. The sample size required for each group was ≥45 cases on the basis of Fisher exact probability.

### Ethical approval:

The study was approved by the Institutional Ethics Committee of Baoding Hospital, Beijing Children’s Hospital Affiliated to Capital Medical University (No.: 2019-22; dated: November 25, 2019) and written informed consent was obtained from all participants’ guardian.

### Inclusion criteria:

- Children who met the diagnostic criteria for IM in the 8th edition of Zhufutang Practical Pediatrics.
- Children who were positive for Epstein-Barr (EB) virus antibody.
- Aged 3 ~ 14 years.
- Parents of children who voluntarily participated in the study and signed the informed consent form.

### Exclusion criteria:

- Children with connective tissue disease or immunodeficiency disease or tumor.
- Children with other viral infections.
- Children with long-term use of corticosteroids or gamma globulin given in the past two months.
- Children allergic to ganciclovir and interferon.

Children in both groups were given basic treatment: adequate rest, active supplementation of vitamins, energy support, abatement of fever, liver protection, etc. The control group was treated with ganciclovir (Weisi, Yangtze River Pharmaceutical Group). Ganciclovir 5 mg/(kg·d) was added to 1 L/g 0.9% sodium chloride solution and then diluted for intravenous drip (≥1 h, once/12 h, lasting 10-14 days. In the meantime, the observation group was treated with human interferon α-1b (Yundesu, Beijing Tri-Prime Gene Pharmaceutical Co., Ltd.) in addition to the treatment given to the control group, with the same dosage of ganciclovir used as the control group. The usage of human interferon α-1b: 1-2 μg/kg was dissolved in 2 ML 0.9% sodium chloride injection and then inhaled through oxygen-driven atomization, 15 min/time, twice/day, lasting seven days.

### Outcome Indicators and Efficacy Evaluation Criteria:

Peripheral blood samples were collected from the two groups pre-treatment (within 24 hour after admission) and 10 days post-treatment. Humoral immune indicators: three ml of fasting blood was collected from the subjects in the morning and centrifuged at 3,000 r/min, followed by grouping. Afterward, the upper plasma was extracted, with humoral immune indicators in the serum, including immunoglobulin G(IgG) and immunoglobulin A(IgA), measured through radioimmunoassay. In the meantime, cytometry was performed using flow cytometry, including CD3+, CD4+, CD4+/CD8+. During the study, physical examinations were performed by the same physician, and manifestation such as the abatement of fever, cervical lymphadenopathy, disappearance time of hepatosplenomegaly, and the length of hospital stay were recorded. At present, the follow-up time was three months. Moreover, the occurrence of adverse reactions was observed, including the number and outcome of children with gastrointestinal reactions such as nausea and vomiting, thrombocytopenia, and rash.

### Statistical Methods

SPSS 22.0 software was used for statistical analysis. Measurement data were expressed as (±S), and the independent sample t-test was utilized for data analysis between the observation and control groups. The comparative analysis of all indicators in the two groups pre- and post-treatment was performed using the paired t-test. Count data were expressed as percentages (%), and the inter-group comparison was conducted using the χ^2^ test. P< 0.05 was considered statistically significant.

## RESULTS

The observation group: 22 males and 23 females, aged 7-13 years, with a mean age of (9.09 ± 1.83) years; fever (n=33), tonsillitis (n=28), rash (n=21), lymphadenopathy (n=36), and hepatosplenomegaly (n=28). The control group: 23 males and 22 females, aged 5-13 years, with a mean age of (9.22 ± 1.73) years; fever (n=32), tonsillitis (n=30), rash (n=21), lymphadenopathy (n=35), and hepatosplenomegaly (n=30). No significant differences were observed in the general data between the two groups (p> 0.05).

### Comparison of pre-treatment indicators between the two groups:

No significant difference was observed in the immune factors IgG, IgA, CD3+, CD4+, and CD4+/CD8+ values between the observation group and the control group (p> 0.05). Comparison of pre- and post-treatment indicators between the two groups: IgG, IgA, CD4+, and CD4+/CD8+ values post-treatment were significantly higher than pre-treatment values, while CD3+ values were markedly lower than pre-treatment values, with statistically significant differences (p< 0.05), indicating effective treatments in both groups of children.

### Comparison of post-treatment indicators between the two groups:

IgG, IgA, CD4+, and CD4+/CD8+ values in the observation group were significantly elevated, of which IgA values in the observation group were higher than that in the control group, and the difference was statistically significant (p< 0.05). Moreover, CD3+ values in the observation group decreased considerably and were evidently lower than in the control group, with significant differences (p< 0.05) Table-I. The time to fever subsidence, the time to cervical lymphadenopathy subsidence, and the time to hepatosplenomegaly subsidence in the observation group were shorter than in the control group, with no significant differences(p>0.05). However, the observation group experienced shorter hospital stays than the control group, and the difference was significant (p<0.05) Table-II.

**Table-I:** Comparative analysis of pre- and post-treatment indicators in the observation group (*χ̅*±*S*) n=45.

Group	IgG		t	P	IgA		t	P
	Pre-treatment	Post-treatment			Pre-treatment	Post-treatment		
Observation	7.47±0.80	11.94±1.19	22.19	0.00	0.91±0.21	1.50±0.41	7.97	0.00
Control	7.56±1.03	9.96±0.80	16.21	0.00	0.77±0.20	1.14±0.16	8.33	0.00
*t*	0.48	9.20			3.10	5.52		
*P*	0.14	0.03			0.47	0.00		

**Table:** Comparative analysis of pre- and post-treatment indicators in the observation group (*χ̅*±*S*)n=45.

	CD3+		t	P	CD4+		t	P	CD4+/CD8+		t	P
	Pre- Treatment	Post- treatment			Pre- treatment	Post- treatment			Pre-treatment	Post-treatment		
Observation	81.16±7.99	59.73±6.31	15.57	0.00	24.51±1.95	30.49±2.67	11.43	0.00	0.36±0.05	1.34±026	24.51	0.00
Control	69.8±7.50	61.80±3.03	7.28	0.00	24.53±2.42	28.64±1.95	8.36	0.00	0.36±0.06	1.17±0.25	20.31	0.00
*t*	6.94	1.97			0.05	3.73			0,02	3.18		
*P*	0.46	0.00			0.09	0.11			0.34	0.38		

**Table-II:** Comparison of symptom subsidence between the 2 groups (*χ̅*±*S*)n=45.

Group	Time to fever subsidence(d)	Time to cervical lymphadenopathy subsidence(d)	Time to hepatosplenomegaly subsidence(d)	Length of hospital stay(d)
Observation	5.02±0.78	5.96±0.97	6.29±0.81	10.56±1.23
Control	7.18±0.96	8.00±0.87	8.44±0.94	12.89±1.00
*t*	11.67	10.44	11,60	9.83
*P*	0.17	0.67	0.13	0.04

### The observation group:

six children with adverse reactions, manifested as nausea, vomiting, and other gastrointestinal reactions (n=2), thrombocytopenia (n= 2), and rash (n=2); the control group: five children with adverse reactions, including gastrointestinal reactions(n=2), thrombocytopenia (n=1), and rash (n=2). There was no significant difference in the incidence of adverse reactions between the two groups (χ^2^= 0.104, P= 0.748, p> 0.05). One day after drug withdrawal, the two groups didn’t exhibit evident gastrointestinal reactions, and reexamination through blood routine tests three days after drug withdrawal showed that their platelet returned to normal without hemorrhagic tendency. Additionally, all rash subsided without pigmentation three days after drug withdrawal. No children in either group experienced severe adverse effects Table-III.

**Table-III:** Comparison of adverse drug reactions between the 2 groups[n(%)].

Group	n	Gastrointestinal reactions	Thrombocytopenia	Rash	Adverse effects
Observation	45	2(4.44)	2(4.44)	2(4.44)	6(13.33)
Control	45	2(4.44)	1(2.22)	2(4.44)	5(11.11)
*χ* ^2^					0.104
*P*					0.748

## DISCUSSION

The findings of this study suggested that the medication regimens applied in the two groups were effective and the incidence of adverse reactions was not significantly different, all of which were relieved after drug withdrawal. Specifically, the post-treatment values of immune indicators IgG, IgA, CD4+, and CD4+/CD8+ in the observation group were significantly elevated compared with those in the control group, of which IgA values in the observation group was higher than in the control group, with significant differences (p< 0.05).

Meanwhile, CD3+ values in the observation group decreased significantly, markedly lower than in the control group, and the difference was significant (p< 0.05). Additionally, the time to fever subsidence, the time to cervical lymph node shrinkage, the time to hepatosplenomegaly subsidence, and the length of hospital stay in the observation group were shorter than in the control group, of which the length of hospital stay was considerably shorter, with significant differences (p< 0.05). Therefore, this study further validates that human interferon α-1b combined with ganciclovir can contribute to improving the body’s immune capacity in children with IM, in which IgA and CD3+, the sensitive experimental indicators, are of some clinical significance in evaluating effects for IM, which are consistent with previous research.^6^

Since IgG and IgA are activated, CD3+ is significantly elevated, and CD4+ is decreased asccordingly when the immune function is impaired, the changes in these detection indicators can effectively determine the status of the body’s immune function.^7^-^9^ Infectious mononucleosis (IM) is a prevalent infectious disease in children, primarily caused by Epstein-Barr virus (EBV) infection and characterized by a rapid onset, rapid disease progression, and strong infectivity, which can exhibit a severe impact on the physical development of the affected children.^10^,^11^ Particularly, EBV can be transmitted through droplets, saliva, close contact, and other means, and remains latent in B lymphocytes after invading the oropharyngeal epithelial cells.^12^

Due to the non-fully developed bodily functions of the pediatric population and their lower immune function compared to adults, the excessive release of inflammatory mediators due to IM can gradually disrupt their immune system function and affect their immunity when their immune function is impaired or decreased^13^, ultimately leading to the inability to effectively resist the viral invasion and an aggravation of the disease.^14^ As one of nucleotide broad-spectrum anti-DNA viral drugs, ganciclovir is commonly used in the clinical treatment of IM and can compete to inhibit the binding of trivalent phosphate of deoxyguanosine to DNA polymerase, terminate DNA strand elongation, block viral DNA replication, transcription, and exert antiviral effects, thereby controlling disease progression and relieving clinical manifestation.^15^-^17^ With the wide application of ganciclovir in clinical practice, it has been found that some children may experience disease recurrence and unsatisfactory prognosis.^18^ Currently, no specific drug is available for IM treatment.

Therefore, it is of great importance to further explore the best therapy to control the disease, improve the immune function of children with IM, and enhance the efficacy. Human interferon α-1b is a bioactive cytokine, which, after atomization inhalation, can increase the local drug concentration and effect, inhibit intracellular viral replication by inducing cells to produce a variety of antiviral proteins. At the same time, it can also strengthen the function of T lymphocytes and B lymphocytes, regulate the phagocytic process, and promote the improvement of the immune capacity of the body, thereby inhibiting the spread of infected cells.^19^-^21^

### Limitations

However, this study also has some shortcomings, such as a small number of patients were included and no long-term follow-up was conducted. In view of this, more samples should be included and follow-up time should be increased in future studies to further validate the findings of this study.

## CONCLUSIONS

Compared with ganciclovir alone, human interferon α-1b combined with ganciclovir in the treatment of IM can lead to faster subsidence of clinical manifestation, improved immune capacity of the body, and shorter hospital stay, thereby making it worthy of promotion.

### Authors’ Contributions:

**JZ and HS:** Literature search**,** designed the study, and are responsible and accountable for the accuracy or integrity of the work.

**JW and JB:** Collected and analyzed the data; Critical Review.

**YZ:** Drafted the article and revised it critically for important intellectual content.

All the authors have read and agreed to the final version of the manuscript.
